# Indirubin-3′-oxime stimulates chondrocyte maturation and longitudinal bone growth via activation of the Wnt/β-catenin pathway

**DOI:** 10.1038/s12276-019-0306-3

**Published:** 2019-09-12

**Authors:** Sehee Choi, Pu-Hyeon Cha, Hyun-Yi Kim, Kang-Yell Choi

**Affiliations:** 1 Translational Research Center for Protein Function Control, Yonsei University, Seoul, Korea; 2 Department of Biotechnology, College of Life Science and Biotechnology, Yonsei University, Seoul, Korea; 3 CK Biotechnology Inc., Rm 417, Engineering Research Park, 50 Yonsei Ro, Seodaemun-Gu, Seoul, 03722 Korea

**Keywords:** Bone development, Molecularly targeted therapy

## Abstract

Researchers have shown increased interest in determining what stimulates height. Currently, many children undergo precocious puberty, resulting in short stature due to premature closure of the growth plate. However, the current approach for height enhancement is limited to growth hormone treatment, which often results in side effects and clinical failure and is costly. Although recent studies have indicated the importance of paracrine signals in the growth plate for longitudinal bone growth, height-stimulating agents targeting the signaling pathways involved in growth plate maturation remain unavailable in the clinic. The Wnt/β-catenin pathway plays a major role in the maturation of growth plate chondrocytes. In this study, by using an ex vivo tibial culture system, we identified indirubin-3′-oxime (I3O) as a compound capable of enhancing longitudinal bone growth. I3O promoted chondrocyte proliferation and differentiation via activation of the Wnt/β-catenin pathway in vitro. Intraperitoneal injection of I3O in adolescent mice increased growth plate height along with incremental chondrocyte maturation. I3O promoted tibial growth without significant adverse effects on bone thickness and articular cartilage. Therefore, I3O could be a potential therapeutic agent for increasing height in children with growth retardation.

## Small molecular approach to increase height growth

A compound that stimulates longitudinal bone growth could lead to safer treatments for children with short stature. Growth hormone treatments can normalize development in some children with growth hormone deficiency, but the side effects can be severe. Researchers led by Kang-Yell Choi at Yonsei University, Seoul, South Korea, have determined that chemical stimulation of a critical cell signaling pathway involving bone growth may offer a better approach for growing taller. Longitudinal growth of bones is driven by the proliferation and differentiation of cartilage cells in the growth plate. Choi and colleagues screened a chemical library and identified a compound derived from traditional Chinese herbs, which efficiently promotes this bone growth process in cultured cartilage cells and in the tibias of three-week-old mice. This compound appears safe, suggesting a potentially better avenue for promoting height growth.

## Introduction

Height growth occurs rapidly in childhood and eventually ceases during puberty^1^. Therefore, approaches promoting height at the period before growth plate closure occurs are important. The current therapies for treating children with short stature include administration of growth hormone (GH) alone or together with a gonadotropin-releasing hormone (GnRH) analog^2^; however, studies have revealed that the efficacy of these therapies is restricted to a minority with GH deficiency^3^. Furthermore, these treatments can result in harmful side effects, such as hyperinsulinism edema, pseudotumor cerebri, and gynecomastia^4^. Therefore, alternative therapeutic strategies for enhancing height are an unmet need in drug development.

Longitudinal bone growth is the result of chondrocyte proliferation and hypertrophic differentiation at the growth plate followed by conversion of their cartilage intermediates into new bone; this process is called endochondral ossification. In the past, childhood growth was thought to be regulated by endocrine factors, such as GH, thyroid hormone, estrogen, androgen, vitamin D, and glucocorticoids, entering the circulation. However, recent studies of mutations in human skeletal dysplasias and genetic manipulation have revealed that paracrine factors acting locally in the growth plate and intracellular molecular pathways are important for chondrocyte maturation and longitudinal bone growth^3,5,6^. Furthermore, circulating endocrine factors have been demonstrated to exert their effects on growth plate chondrocytes through activation of the local signaling pathway within the growth plate^7,8^.

The Wnt/β-catenin pathway has emerged as a critical route for longitudinal bone growth involved in chondrocyte maturation and cartilage development in vitro and in vivo. Cartilage-specific deletion of *Ctnnb1* encoding β-catenin resulted in decreased proliferation and differentiation of chondrocytes at the growth plate^9–11^. Lipoprotein receptor-related protein 4 (*Lrp4*)-deficient mice showed growth retardation in their limbs^12^. Additionally, inactivation of glycogen synthase kinase 3 (GSK3), which results in β-catenin destabilization, increased longitudinal growth of ex vivo cultured tibia^13,14^. Taken together, these results suggested that activation of the Wnt/β-catenin pathway can be a therapeutic approach to increase longitudinal bone growth. However, there are no clinically available height-stimulating agents targeting the Wnt/β-catenin pathway.

In this study, we examined the effects of small molecular activation of the Wnt/β-catenin pathway on longitudinal bone growth in tibia cultured ex vivo. A GSK3β inhibitor, indirubin‐3′‐oxime (I3O), showed the most dramatic increase in bone length and stimulated the proliferation and differentiation of chondrocytes. The ability of I3O to promote longitudinal bone growth was also demonstrated in vivo. Specifically, increases in tibial length with growth plate activation were observed in adolescent mice. Overall, our findings suggest that I3O could be a potential agent for promoting height growth in children with growth retardation.

## Materials and methods

### Cell culture and reagent treatment

The mouse chondrogenic cell line ATDC5 was obtained from the RIKEN Cell Bank (Ibaraki, Japan). Rat chondrosarcoma RCS cells were provided by Dr. D.W. Kim (Yonsei University, Korea). ATDC5 cells were maintained in DMEM/F12 (1:1) (Gibco, Grand Island, NY) supplemented with 5% FBS (Gibco). To induce hypertrophic differentiation, the ATDC5 cells were incubated with insulin-transferrin-sodium selenite (ITS) supplement (Gibco) in three-dimensional alginate beads for 3 days as described previously^15^. RCS cells were maintained in DMEM (Gibco) containing 10% FBS. All chemicals were dissolved in dimethyl sulfoxide (DMSO; Sigma-Aldrich, St. Louis, MO) for the in vitro studies.

### Tibial organ culture

Tibias were isolated from embryonic day 15.5 (e15.5) mice and cultured for 6 days in serum-free α-MEM (Gibco) containing ascorbic acid, β-glycerophosphate, BSA, l-glutamine, and penicillin–streptomycin as described previously^14^. After dissection, tibias were incubated in medium overnight and then treated with chemicals. The medium and reagents were changed every 48 h. Tibial images were captured using a SMZ-745T microscope (Nikon, Tokyo, Japan). Tibial length was measured prior to treatment and after 6 days in culture. The samples were then prepared for paraffin embedding, sectioned, and analyzed by immunohistochemistry.

### Plasmids, siRNAs, and transfection

The pGL3-Col10a1 promoter was provided by H. Kawaguchi^15^. pGL3-Col10a1 and pTOPFLASH were used for luciferase assays with the pCMV–β-galactosidase reporter (Clontech).

The following siRNA sequences were used for ATDC5 cells: *Ctnnb1* siRNA-1, sense AUUACAAUCCGGUUGUGAACGUCCC and antisense GGGACGUUCACAACCGGAUUGUAAU, *Ctnnb1* siRNA-2, sense UAAUGAAGGCGAACGGCAUUCUGGG, and antisense CCCAGAAUGCCGUUCGCCUUCAUUA

Lipofectamine (Invitrogen, Carlsbad, CA) was used for plasmid transfection, and RNAiMax (Invitrogen) was used for siRNA transfection, according to the manufacturer’s instructions.

### Mouse models

Three-week-old C57BL/6 mice were obtained from KOATECH (Gyeonggido, Korea). To investigate the effects of I3O treatment on longitudinal bone growth, I3O (0.05 or 0.5 mg/kg) was administered daily by intraperitoneal (i.p.) injection for 2 or 10 weeks. For BrdU-labeling experiments, mice were intraperitoneally injected with 50 mg/kg BrdU (Sigma Aldrich) before 24 and 2 h prior to sacrifice. All experiments were approved by the Institutional Animal Care and Use Committee (IACUC) of Yonsei University (Korea) and conducted based on the guidelines of the Korean Food and Drug Administration.

### Radiological and histochemical analyses

Plain radiographs were taken using an X-ray apparatus (KODAK DXS 4000 Pro SYSTEM; Carestream Health, Rochester, NY). The tissues were fixed in 4% paraformaldehyde (PFA), decalcified in 10% EDTA (pH 7.4), dehydrated, embedded in paraffin, and sectioned to 4 μm thickness (Leica Microsystems, Wetzlar, Germany). The tissue sections were rehydrated and used for further analyses, including H&E, TRAP, and immunohistochemical (IHC) staining. To perform IHC analysis, the sections were incubated with citrate buffer (pH 6.0) at 80 °C for 30 min or with 0.5% pepsin (Sigma-Aldrich) at 37 °C for 15 min. Then, the sections were blocked with 5% normal goat serum (NGS; Vector Laboratories, Burlingame, CA) and 0.3% Triton-X-100 in PBS at room temperature for 1 h. For 3,3′-diaminobensidine (DAB) staining, the sections were incubated with 0.345% H_2_O_2_ for 15 min. Before incubating the sections with mouse primary antibody, mouse IgG was blocked using a M.O.M kit (Vector Laboratories). The sections were incubated at 4 °C overnight with the following primary antibodies: anti-BrdU (Sigma) and anti-β-catenin (BD Bioscience, San Jose, CA, #610154; 1:50). Then, the sections were incubated at room temperature for 1 h with biotinylated anti-mouse (Vector Laboratories, BA-9200; 1:200) or biotinylated anti-rabbit (Vector Laboratories, BA-1000; 1:200) secondary antibodies. The sections were then incubated in avidin–biotin complex solutions (Vector Laboratories), stained with a DAB kit (Vector Laboratories) for 3−30 min, and counterstained with methyl green (Sigma-Aldrich). All incubations were conducted in humid chambers. Staining was observed with an ECLIPSE TE2000-U microscope (Nikon). For fluorescence staining, the sections were incubated with anti-collagen 2a1 (Thermo Fisher Scientific, MA, PA5-11462; 1:100) at 4 °C overnight, followed by incubation with anti-rabbit Alex Fluor 555 (Thermo Fisher Scientific, A21428; 1:200) secondary antibodies at room temperature for 1 h. The sections were then counterstained with DAPI (Sigma-Aldrich) for 5 min and mounted in Gel/Mount media (Biomeda Corporation). All incubations were conducted in dark, humid chambers. The fluorescence signal was visualized using a LSM700 META confocal microscope (Carl Zeiss Inc., Thornwood, NY) at excitation wavelengths of 543 nm (Alexa Fluor 555) and 405 nm (DAPI).

### Immunocytochemistry

ATDC5 cells were seeded on glass coverslips in 12-well culture plates. The cells were washed with PBS and fixed with 4% PFA at room temperature for 15 min. After permeabilization with 0.1% Triton-X-100 for 15 min and blocking with 5% BSA for 1 h, the cells were incubated with primary antibody specific for β-catenin (1:100) at 4 °C overnight. The cells were washed in PBS and incubated with Alexa Fluor 488 secondary antibody (1:200) at room temperature for 1 h. Cell nuclei were counterstained with DAPI for 10 min, and the stained samples were examined under a LSM700 META microscope using 405 or 488-nm excitation wavelengths.

### Immunoblot analyses

Whole cell extracts were prepared by lysing the cells with RIPA buffer (150 mM NaCl, 50 mM Tris, pH 7.4, 1% NP-40, 0.25% sodium deoxycholate, 1 mM EDTA, protease inhibitors, and phosphatase inhibitors). Protein samples were separated by 8–12% sodium dodecylsulfate (SDS) polyacrylamide gel and transferred to a nitrocellulose membrane (Whatman). The membranes were blocked and then incubated with the following primary antibodies: anti-β-catenin (Santa Cruz Biotechnology, Santa Cruz, CA, sc-7199; 1:3000), anti-p-GSK3β (S9; Cell Signaling Technology, #9336S; 1:1000), and anti-α-tubulin (Cell Signaling Technology, #3873S; 1:20,000). Horseradish-peroxidase-conjugated anti-mouse (Cell Signaling Technology, #7076; 1:3000) or anti-rabbit (Bio-Rad, #1706515; 1:3000) secondary antibodies were used. The blots were visualized by an enhanced chemiluminescence (ECL) detection system using a luminescent image analyzer, LAS-3000 (Fujifilm, Tokyo, Japan). Immunoblot bands were analyzed using Multi-Gauge V3.0 software (Fujifilm).

### Cell proliferation assay

ATDC5 cells were seeded in 96-well culture plates and then treated with I3O for 2 days. Next, 0.25 mg/ml of MTT(3-(4,5-dimethylthiazol-2-yl)-2-5-diphenyltetrazolium bromide) (Amresco, solon, OH) was added to each well. After incubation at 37 °C for 2 h, insoluble purple formazan was obtained by removing the medium. The formazan was dissolved in 200 μl of DMSO for 1 h and then analyzed by reading the absorbance at 590 nm.

### Alcian blue staining

RCS cells were seeded in 12-well culture plates and then grown in medium containing I3O for 3 days. The cells were washed with PBS and fixed with 4% PFA at room temperature for 15 min and then stained with 1% Alcian blue 8GS (Sigma) in 5% acetic acid solution.

### RNA extraction and quantitative real-time PCR

Total RNA was extracted using TRIzol reagent (Invitrogen) according to the manufacturer’s instructions. 2 μg of RNA was reverse-transcribed using 200 units of reverse transcriptase (Invitrogen) in a 40-μl reaction carried out at 37 °C for 1 h. For quantitative real-time PCR analyses (qRT-PCR), the resulting cDNA (1 μl) was amplified in 10 μl reaction mixture containing iQ SYBR Green Supermix (Qiagen, Germantown, MD) and 10 pmol of the primer set (Bioneer). The comparative cycle-threshold (CT) method was used, and *Actb* encoding β-actin served as an endogenous control. The primer sets are shown in Table 1.

**Table 1:** qPCR primer sets

Gene	Strand	Primer sequences
*Actb*	F	5′-GGATGCAGAAGGAGATTACT-3′
	R	5′-CCGATCCCACACAGAGTACTT-3′
*Alp*	F	5’-GGGACTGGTACTCGGATAAC-3′
	R	5’-CTGATATGCGATGTCCTTGC-3′
*Col2a1*	F	5′-GCCTGTCTGCTTCTTGTAA-3′
	R	5′-TGCGGTTGGAAAGTGTTT-3′
*Col10a1*	F	5′- TCCACTCGTCCTTCTCAG-3′
	R	5′-TTTAGCCTACCTCCAAATGC-3′
*Ctnnb1*	F	5′-ACAAGCCACAAGATTACAAGAA-3′
	R	5′-GCACCAATATCAAGTCCAAGA-3′
*Mmp9*	F	5′-TGAAGTCTCAGAAGGTGGAT-3′
	R	5′-ATGGCAGAAATAGGCTTTGT-3′
*Mmp13*	F	5′-TAAGACACAGCAAGCCAGA-3′
	R	5′-CACATCAGTAAGCACCAAGT-3′
*Runx2*	F	5′-AAGGACAGAGTCAGATTACAGA-3′
	R	5′-GTGGTGGAGTGGATGGAT-3′
*Sox9*	F	5′-AACTGGAAACCTGTCTCTCT-3′
	R	5′-ACAACACACGCACACATC-3′
*Vegfa*	F	5′-TTATTTATTGGTGCTACTGTTTATCC-3′
	R	5′-TCTGTATTTCTTTGTTGCTGTTT-3′

*F* forward, *R* reverse

### μCT analyses

To analyze the trabecular and cortical bone of the tibia, standardized cone-beam microcomputed tomography (μCT) scanning was performed using a μCT system for small animal imaging (Skyscan 1076; Skyscan, Kontich, Belgium). The scanned image data were reconstructed to create 3D images and analyzed using the CT-Analyzing Program (Skyscan). Acquisition settings were as follows: X-ray source voltage, 70 kVp; current, 140 μA; 0.5-mm-thick aluminum filter was used for beam hardening reduction; pixel size, 18 μm; exposure time, 14.7 s; and rotation step, 0.5° with a complete rotation over 360°.

### Statistics

All data are presented as the mean ± s.e.m., and the number of samples is indicated in each figure legend. Statistical significance was determined by Student’s *t*-test as indicated in the figures; *P < 0.05; **P < 0.005; ***P < 0.0005. The results shown are representative of at least three independent experiments.

## Results

### Identification of small molecules that increase ex vivo tibial growth and activate Wnt/β-catenin signaling

**Fig. 1:**
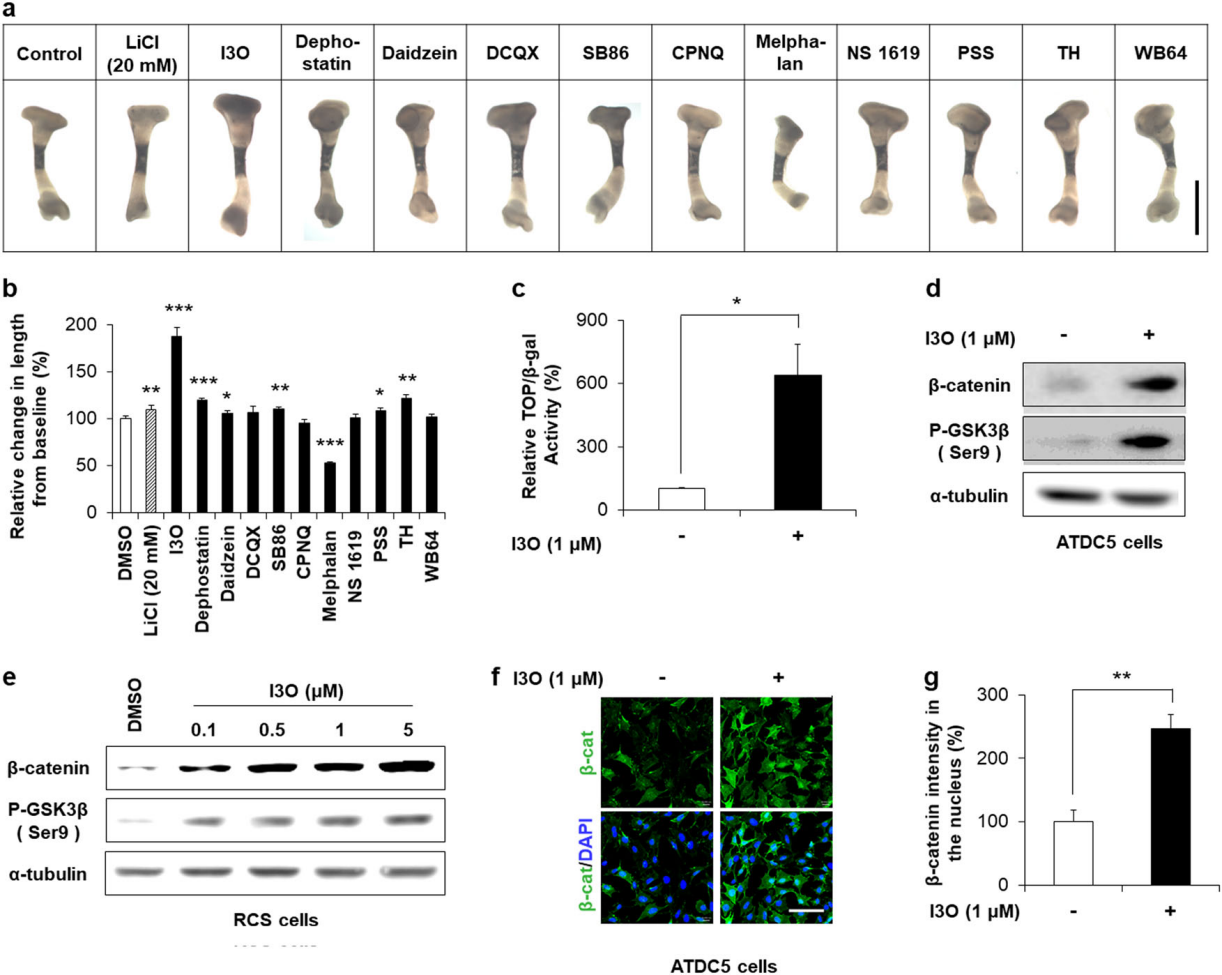
Identification of I3O as an enhancer for the elongation of ex vivo cultured tibia. **a** and **b** Tibias isolated from E15.5 mice were cultured ex vivo for 6 days with 5 μM of each small molecule. LiCl (20 mM) was used as a positive control for activation of the Wnt/β-catenin pathway. Representative images of tibias after 6 days of culture with the indicated small molecules **a**. Scale bar, 1 mm. Longitudinal growth of tibias over 6 days of culture (the difference between the beginning and end of culture) was quantified (mean ± s.e.m., *n* = 3 per group, *P < 0.05, **P < 0.005, and ***P < 0.0005 versus DMSO-treated control) **b**. **c** For the luciferase reporter assay, ATDC5 cells cotransfected with pTOPFlash and pCMV-β-gal were treated with I3O for 24 h (mean ± s.e.m., *n* = 2 per group, *P < 0.05). **d–g** ATDC5 or RCS cells were treated with I3O for 24 h, followed by immunoblotting to detect β-catenin, GSK3β, and α-tubulin **d**, **e** and immunofluorescence staining to visualize β-catenin **f**. Scale bar, 0.1 mm. The intensities of β-catenin were analyzed from the immunofluorescence staining images using NIS Elements V3.2 software (mean ± s.e.m., *n* = 3 per group, **P < 0.005) **g**

We previously screened a pharmacologically active compound library using stable HEK293 cells harboring the TOPFlash reporter gene to identify activators of the Wnt/β-catenin pathway^16^. From these, the top 11 positive compounds were tested using an E15.5 mouse tibial organ culture system to identify stimulators of longitudinal bone growth (Fig. 1a). Seven compounds were found to increase tibia length over the 6 days of ex vivo culture compared with the DMSO-treated control. With an incremental increase of 88% compared to the control, I3O was identified as the compound that most significantly increased tibia length (Fig. 1b).

To evaluate the effects of I3O on activation of the Wnt/β-catenin pathway in chondrocytes, we performed a TOPFlash reporter assay in ATDC5 cells, a murine chondrogenic cell line. I3O markedly increased reporter activity by 538% compared to the control (Fig. 1c). The positive effect of I3O on Wnt/β-catenin pathway activation in chondrocytes was confirmed by immunoblot analyses. Increased β-catenin and phosphorylated GSK3β at serine 9 was observed following I3O treatment in RCS cells, a rat chondrosarcoma cell line, as well as in ATDC5 cells (Fig. 1d, e). The effect of I3O on Wnt/β-catenin signaling activation was also verified by the nuclear accumulation of β-catenin in I3O-treated ATDC cells (Fig. 1f, g).

### I3O increases tibial length in an ex vivo culture system with activation of growth plate chondrocytes

**Fig. 2:**
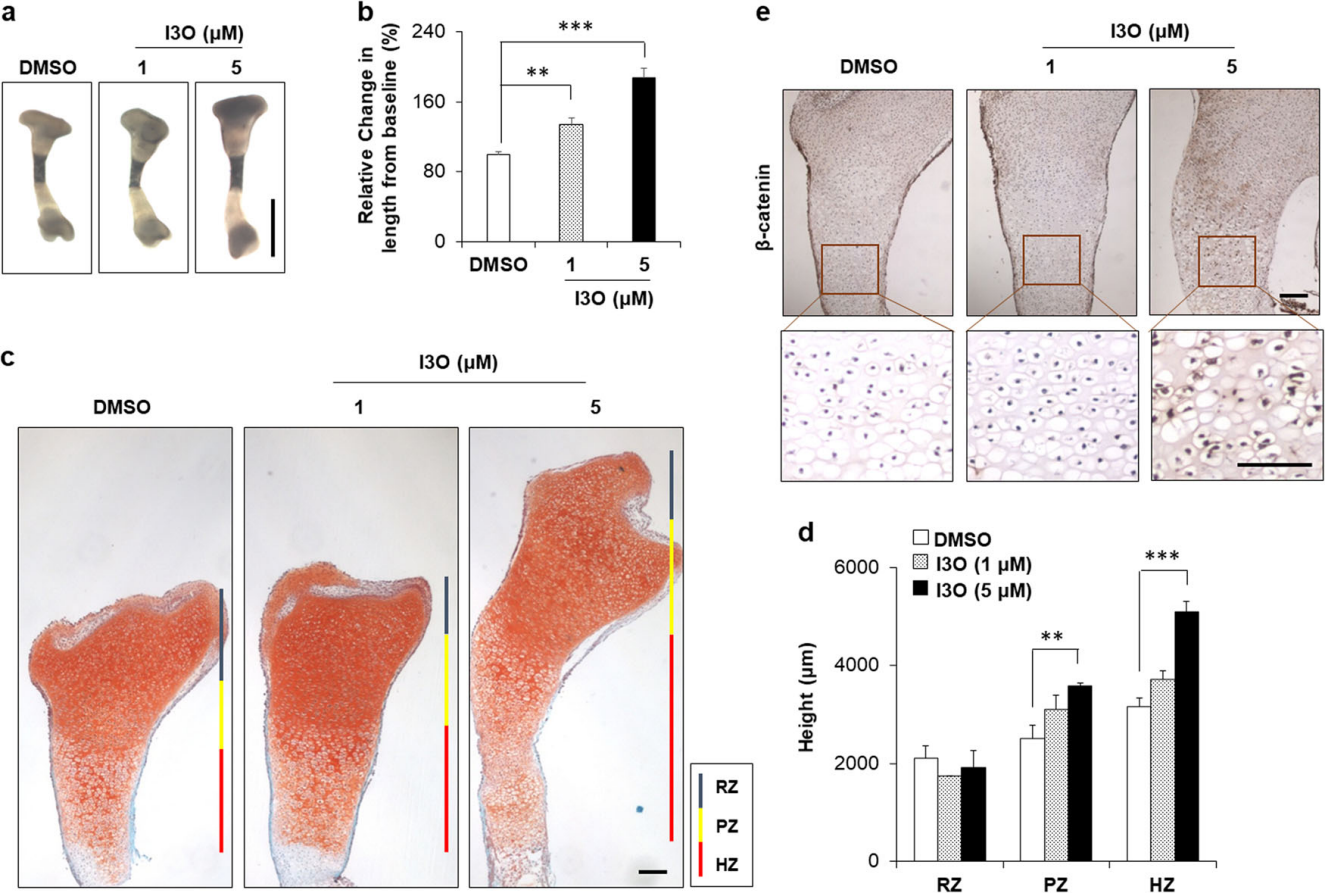
The effects of I3O on the length of the ex vivo cultured tibia. **a–e** Ex vivo tibial cultures (E15.5) were treated with 0, 1, and 5 μM of I3O for 6 days. Representative images of tibias after 6 days of culture are shown **a**. Scale bar, 1 mm. The relative lengths of tibias over 6 days of culture were quantified (mean ± s.e.m., *n* = 3 per group, **P < 0.005 and ***P < 0.0005) **b**. Safranin O-stained paraffin sections of ex vivo cultured tibias were analyzed to compare the morphology and height of growth plate zones, including the resting zone (RZ), proliferating zone (PZ), and hypertrophic zone (HZ) **c**. Scale bar, 0.1 mm. **d** Each zone height in the growth plates of DMSO-treated and I3O-treated tibias was quantified (mean ± s.e.m., *n* = 3 per group, **P < 0.005 and ***P < 0.0005). Immunohistochemical staining for β-catenin in the growth plates of tibias **e**. Scale bars, 0.1 mm

We examined the dose-dependent effects of I3O on increasing tibial length ex vivo (Fig. 2a, b). To assess the cellular state and organization in the growth plate, tibial sections were stained with safranin O to detect cartilage. I3O treatment increased the height of the tibia growth plate in a dose-dependent manner with elevation of the proliferative zone (PZ) from 23% to 43% and the hypertrophic zone (HZ) from 17% to 60% at 1 and 5 μM concentrations, respectively (Fig. 2c, d). These results showed that I3O promotes the transition from proliferative chondrocytes to hypertrophic chondrocytes, as well as from resting chondrocytes to proliferative chondrocytes. Immunohistochemistry analysis revealed that the β‐catenin level was increased in the growth plate of I3O‐treated tibia, especially in the HZ, compared with the DMSO‐treated control (Fig. 2e). Taken together, these results showed that I3O promotes longitudinal bone growth by increasing the overall height of the growth plate with the induction of chondrocyte proliferation and differentiation.

### I3O stimulates chondrocyte maturation via activation of the Wnt/β-catenin pathway

**Fig. 3:**
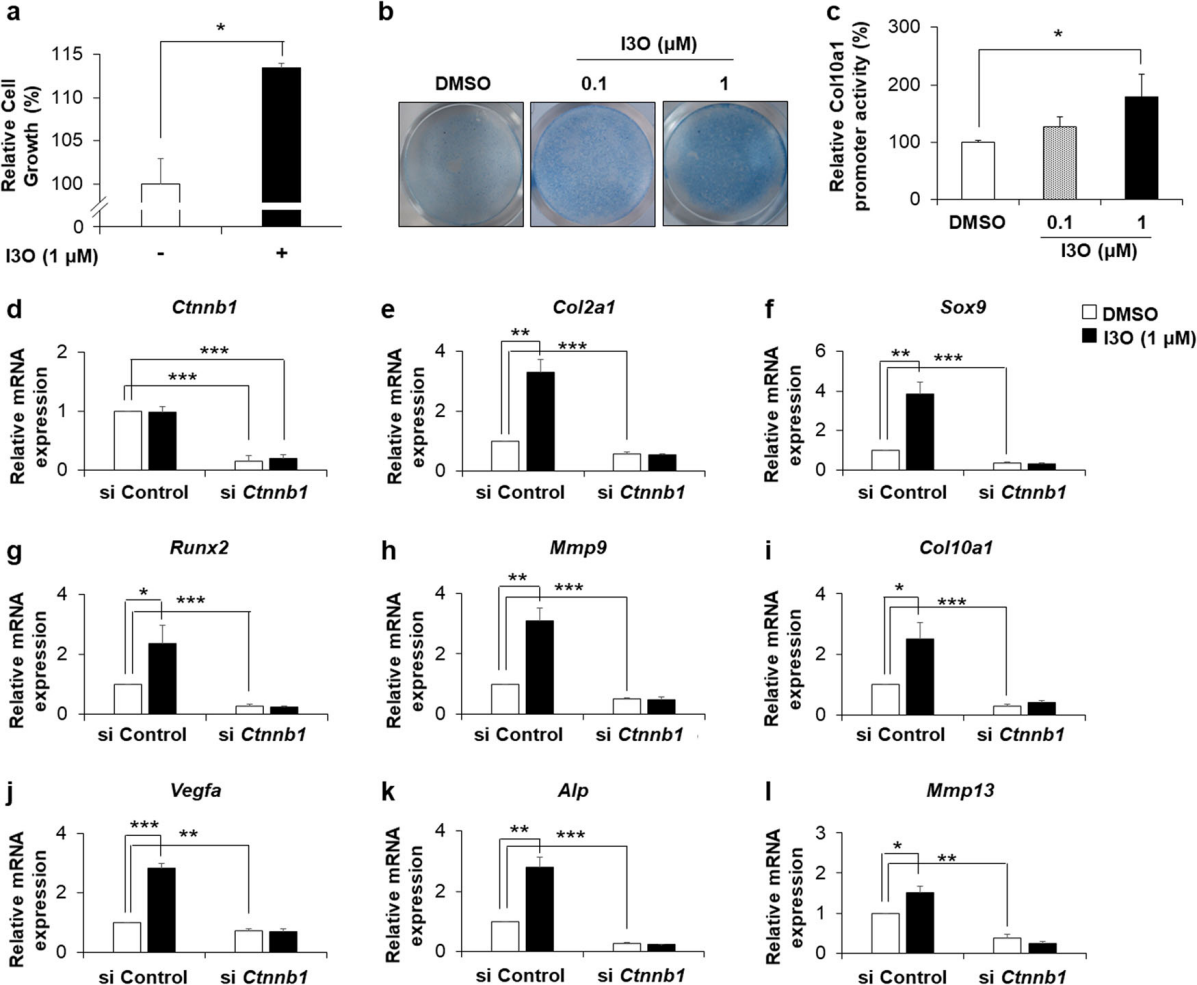
The effects of I3O on chondrocyte proliferation and differentiation via activation of the Wnt/β-catenin pathway. **a** ATDC5 cells were treated with 1 μM of I3O for 48 h, and cell growth was assessed by the 3-(4,5-dimethylthiazol-2-yl)-2,5-diphenyltetrazolium bromide (MTT) assay (mean ± s.e.m., *n* = 3 per group, *P < 0.05). **b** RCS cells treated with DMSO or I3O were subjected to Alcian blue staining. **c** Col10a1 promoter activity was analyzed by treatment with 1 μM I3O in ATDC5 cells transfected with a luciferase reporter gene construct containing a cloned 4.5-kb promoter fragment of COL10 (mean ± s.e.m., *n* = 3 per group, *P < 0.05). **d–l** ATDC5 cells transfected with control siRNA or *Ctnnb1* siRNA were treated with or without 1 μM I3O for 72 h in three-dimensional alginate beads. The relative mRNA levels of chondrogenic differentiation markers were measured by qRT-PCR (mean ± s.e.m., *n* = 3 per group, *P < 0.05, **P < 0.005, and ***P < 0.0005)

We next evaluated the effect of I3O on chondrocyte proliferation and differentiation at the cellular level. I3O increased the cellular growth rate of ATCD5 cells (Fig. 3a). To verify the formation of typical cartilage nodules and the synthesis of matrix proteoglycans, we performed Alcian blue staining of RCS cells. Our results demonstrated that cells treated with I3O enhanced the frequency of stained nodules in a dose-dependent manner (Fig. 3b). In addition, the promoter activity of type X collagen (Col10a1), a representative hypertrophic differentiation marker, was increased by I3O treatment, revealing that I3O directly promotes hypertrophic differentiation of chondrocytes (Fig. 3c). To confirm these results, we analyzed the mRNA expression level of chondrogenic markers in ATDC5 cells cultured in three-dimensional alginate beads in the presence or absence of I3O for 3 days. I3O significantly increased the mRNA levels of early chondrogenesis markers, such as Col2a1 and Sox9. In addition, I3O treatment increased the mRNA levels of fully differentiated hypertrophic chondrocyte markers, such as Col10a1, Vegfa, Alp, and Mmp13, as well as prehypertrophic chondrocyte markers, such as Runx2 and Mmp9 (Fig. 3d−l). Transcriptional induction of these genes by I3O treatment was suppressed by knockdown of *Ctnnb1* (Fig. 3d−l). Collectively, our results showed that I3O induced chondrogenic differentiation via activation of the Wnt/β‐catenin pathway.

### I3O promotes the activation of growth plates in mice

**Fig. 4:**
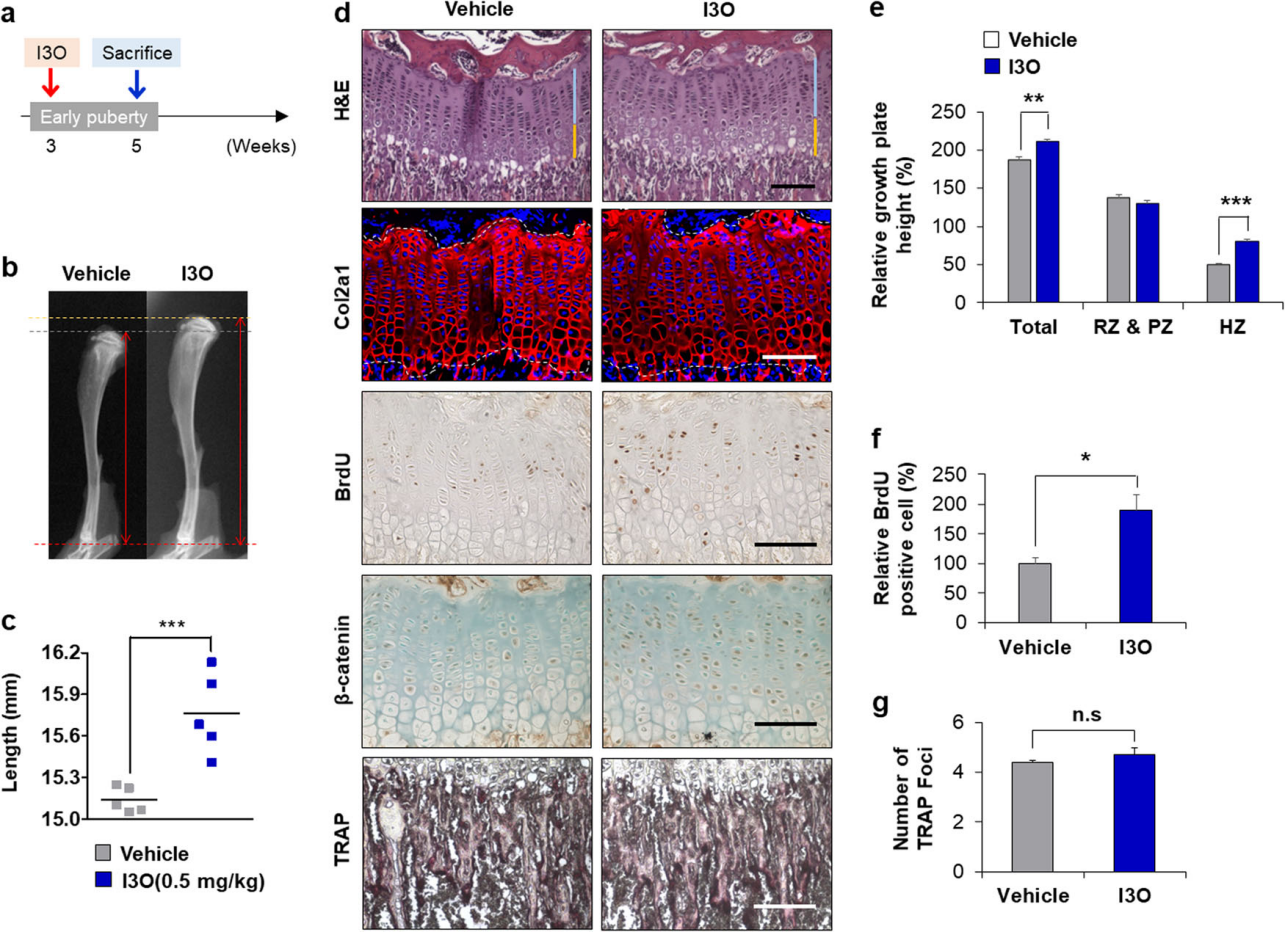
The effects of I3O on the maturation of the mouse growth plate. **a–g** I3O (0.5 mg/kg) was administered to 3-week-old mice daily by intraperitoneal injection for 2 weeks. Schematic diagram of in vivo experiments **a**. Representative radiographs are shown **b** along with the tibial length measurements **c** (mean ± s.e.m., *n* = 5 per group, ***P < 0.0005). The growth plates of proximal tibias treated or untreated with I3O underwent H&E staining, immunohistochemical analyses using the indicated antibodies, and tartrate resistant acid phosphatase (TRAP) staining for the osteoclast marker (dark purple) **d**. The area within the dashed lines indicates the growth plate zone. Scale bars, 0.1 mm. Quantitative analysis of height in the resting and proliferative zones (RZ&PZ) and the hypertrophic zone (HZ) (mean ± s.e.m., *n* = 5 per group, **P < 0.0005) **e**. Quantitative analysis of BrdU-positive cells in growth plates (mean ± s.e.m., *n* = 5 per group, *P < 0.005) **f**. The number of TRAP-positive foci was quantified along the cartilage/bone interface (mean ± s.e.m., *n* = 5 per group) **g**. n.s. indicates non-significance compared with vehicle

To investigate the role of I3O on endochondral ossification in vivo, we intraperitoneally injected I3O into mice every other day from 3 weeks of age until 5 weeks of age (Fig. 4a). I3O-treated mice exhibited longer tibial lengths than vehicle-treated mice (Fig. 4b, c). Histological analyses of the tibial growth plate revealed that chondrocytes were well organized and that the width of the Col2a1-positive area was increased following I3O treatment (Fig. 4d). The HZ height in the growth plate was also increased by I3O treatment compared to that of vehicle-treated mice (Fig. 4e). Using tartrate-resistant acid phosphatase (TRAP) staining, we verified that this height extension in the HZ was not due to delayed cartilage resorption (Fig. 4d, g). In the growth plate/trabecular interface, the numbers of TRAP-positive foci were similar between the vehicle-treated and I3O-treated groups. Although the height of the resting zone (RZ) and PZ did not change following I3O treatment, the frequency of BrdU-positive chondrocytes in the growth plates was found to be higher in I3O-treated mice. Furthermore, the number of β-catenin-positive chondrocytes was increased in the I3O-treated growth plates (Fig. 4f). These results showed that I3O induces chondrocyte proliferation and then promotes conversion from the proliferative state to hypertrophic differentiation in the growth plate.

### I3O enhances tibial longitudinal growth in mice without adverse changes in bone thickness parameters

**Fig. 5:**
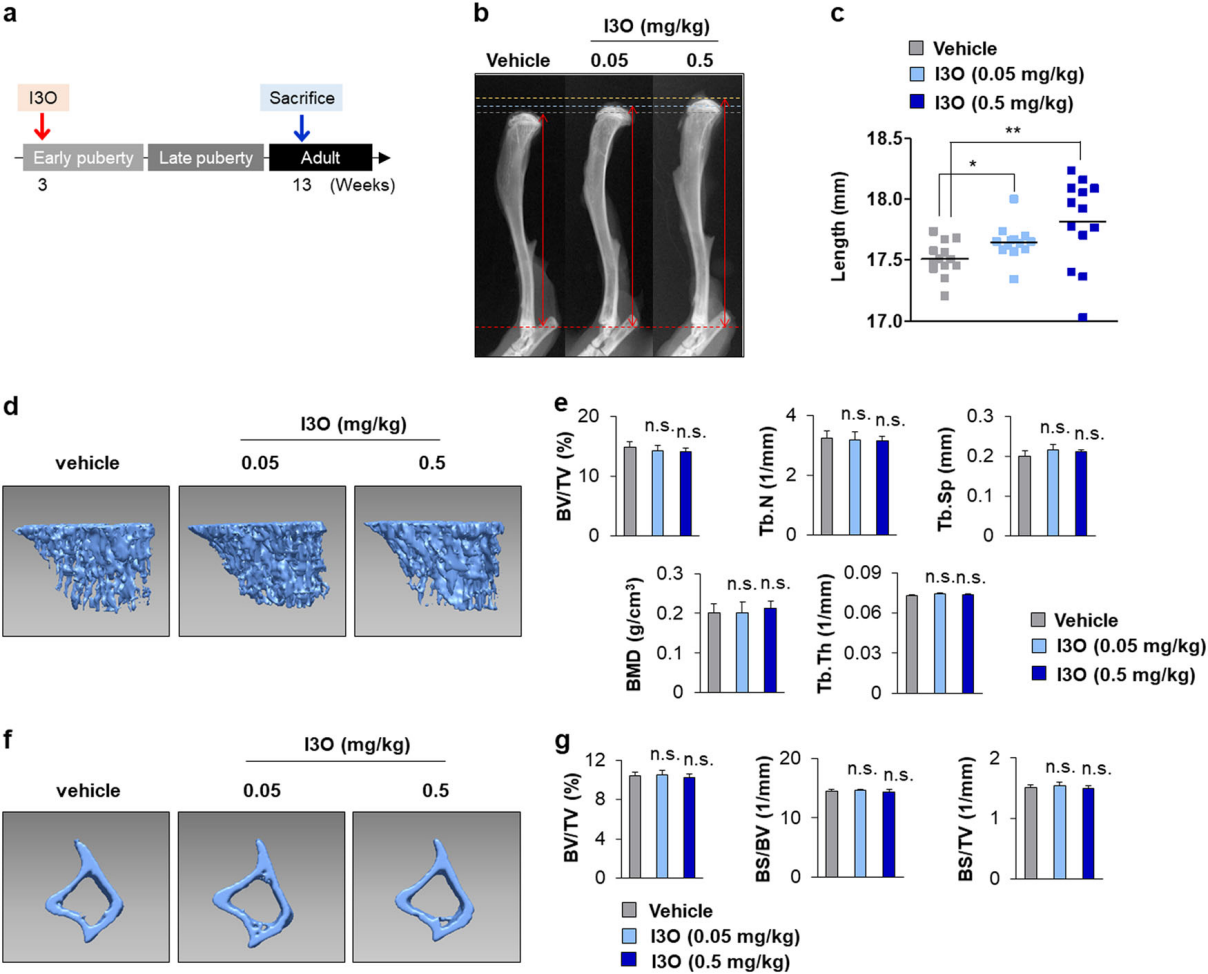
The effects of long-term treatment with I3O on tibia length and bone parameters. **a–g** I3O (0.05 and 0.5 mg/kg) was administered to 3-week-old mice daily by intraperitoneal injection for 10 weeks. Schematic diagram of in vivo experiments **a**. Representative radiographs are shown **b** along with the tibia length measurements (mean ± s.e.m., *n* = 13 per group, *P < 0.05, **P < 0.005) **c**. Representative μCT images of tibial trabecular bones **d** and their parameters, such as bone volume to tissue volume ratio (BV/TV), bone mineral density (BMD), number of trabeculae, and trabecular separation (mean ± s.e.m., *n* = 5 per group) **e**. Representative μCT images of tibial cortical bones **f** and their parameters, such as bone volume to tissue volume ratio (BV/TV), bone surface to bone volume ratio (BS/BV), and bone surface density (BS/TV) (mean ± s.e.m., *n* = 5 per group) **g**. n.s. indicates non-significance compared with vehicle

To determine the effects of I3O on final bone growth, mice at 3 weeks of age were injected daily with I3O for 10 weeks (Fig. 5a). The tibial length of I3O-treated mice increased in a dose-dependent manner (Fig. 5b, c). To test whether the elongated tibia of I3O-treated mice produced adverse effects on bone mass, we analyzed tibia samples using μCT-generated three-dimensional images. Compared with vehicle-treated mice, I3O did not induce any bone loss, as shown by a lack of differences in trabecular bone parameters, including bone volume/tissue volume, trabecular thickness, trabecular number, bone mineral density, and trabecular separation, with I3O treatment (Fig. 5d, e). Moreover, no differences in the cortical bone parameters, including bone volume/tissue volume, bone surface/volume, and bone surface density, were observed between the vehicle-treated and I3O-treated tibia (Fig. 5f, g). Additionally, no adverse effects from I3O were observed on articular cartilage, in which chondrocytes persisted in a stable state. In particular, symptoms related to the osteoarthritis-inducing maturation of chondrocytes into a hypertrophic state were not observed (Supplementary Fig. S1a). The livers of I3O-treated mice did not display any histological abnormalities (Supplementary Fig. S1b). In addition, animal weight between the vehicle-treated and I3O-treated groups did not differ during the 10-week treatment (Supplementary Fig. S1c). Overall, I3O stimulates longitudinal bone growth without adverse changes to bone mass and articular chondrocytes.

## Discussion

Longitudinal bone growth occurs at the growth plate via a multistep process that includes the proliferation and hypertrophic differentiation of chondrocytes followed by ossification. Many recent studies have revealed important roles for paracrine factors, such as IHH, PTHrP, BMPs, WNTs, and FGFs, in growth plate activity and longitudinal bone growth^5,6,17^. Therefore, the signaling pathways involved in regulating these factors have become important potential therapeutic targets for treatment of children with growth retardation^3^. However, the current strategy for the treatment of short stature is restricted to GH therapy even though it is only effective on patients with GH deficiency, which constitute a minority of children with growth retardation^18^. Here, we identified the GSK3β inhibitor I3O as a small molecule enhancing height growth via activation of Wnt/β-catenin signaling in chondrocytes.

The Wnt/β-catenin pathway is a major route for the modulation of longitudinal bone growth. Wnt/β-catenin signaling initially suppresses the differentiation of mesenchymal stem cells into chondrocytes^19–21^. However, once chondrocytes are formed, this signaling cascade promotes the overall process of endochondral ossification required for longitudinal bone growth. Studies of cartilage-specific *Ctnnb1* KO mice revealed that the Wnt/β-catenin pathway plays roles in various processes related to growth plate activation, including chondrocyte proliferation and differentiation^10,22^. Thus, targeting the Wnt/β-catenin pathway could be a feasible strategic approach for the development of height-increasing therapeutic drugs that act on the growth plate itself. In this study, we showed that I3O significantly promoted chondrogenesis and stimulated growth plate activation and tibial elongation through upregulation of the Wnt/β-catenin pathway.

The practical usage of I3O is advantageous because it is an indirubin derivative that is the main active ingredient in a traditional Chinese medicine, Danggui Longhui Wan, used to treat various diseases, including chronic myelocytic leukemia. Furthermore, because I3O was reported to exhibit an antitumor effect^23^, it might decrease the cancer risk that could be caused by aberrant activation of the Wnt/β-catenin pathway. Although activation of the Wnt/β-catenin pathway has been implicated in the pathogenesis of osteoarthritis due to the induction of chondrocyte maturation, we did not observe any degenerative structures in articular cartilage treated with I3O. Thus, determining the appropriate dosage that is effective only for growth plate cartilage, but not articular cartilage, is crucial. We used I3O at a maximum concentration of 0.5 mg/kg in in vivo experiments, in contrast to other studies that used I3O for anti-osteoporosis and anti-obesity treatments at a dose of 10 mg/kg^16,24^. Pharmacological compounds intended to increase bone growth should be administered carefully at the minimal effective concentration to prevent toxicity and other side effects related to degenerative cartilage.

Our study suggests that the Wnt/β-catenin pathway can be used as a potential target for the development of height-promoting therapies. Moreover, with the ex vivo tibial culture system, we provide an easy and fast screening method to identify small molecules that enhance longitudinal bone growth via activation of the Wnt/β-catenin pathway.

Overall, we identified and characterized I3O as a small molecule capable of stimulating chondrocyte proliferation and differentiation in the growth plate via activation of Wnt/β-catenin signaling. I3O, which enhances longitudinal bone growth at low concentrations without detectable toxicity, could be developed as a drug for treating children with growth retardation.

## Supplementary information

Supplementary information.
