# Supplementary material for: Indirubin-3′-oxime stimulates chondrocyte maturation and longitudinal bone growth via activation of the Wnt/β-catenin pathway
Source: Exp Mol Med. 2019 Sep 12;51(9):104. doi: 10.1038/s12276-019-0306-3 (PMC6802626; doi:10.1038/s12276-019-0306-3)
Supplement: Supplementary file 1 — Supplementary information. [file 12276_2019_306_MOESM1_ESM.pdf]

Supplementary Information

Indirubin 3'-oxime stimulates chondrocyte maturation and longitudinal bone growth via activation of the Wnt/β-catenin pathway

Sehee Choi[1,2], Pu-Hyeon Cha[1,2], Hyun-Yi Kim[1,2], Kang-Yell Choi[1,2,3*]

[1]Translational Research Center for Protein Function Control, Yonsei University, Seoul, Korea.

[2]Department of Biotechnology, College of Life Science and Biotechnology, Yonsei University, Seoul, Korea.

[3]CK Biotechnology Inc., Rm 417, Engineering Research Park, 50 Yonsei Ro, Seodaemun-Gu, Seoul 03722, Korea.

* Corresponding author:

Kang-Yell Choi

Department of Biotechnology, College of Life Science and Biotechnology, Yonsei University

50 Yonsei Ro, Seodaemun-Gu

Seoul, Korea 120-749

e-mail: kychoi@yonsei.ac.kr (Tel: +82-2-2123-6592, Fax: +82-2-2123-8284)

Supplementary Figure S1

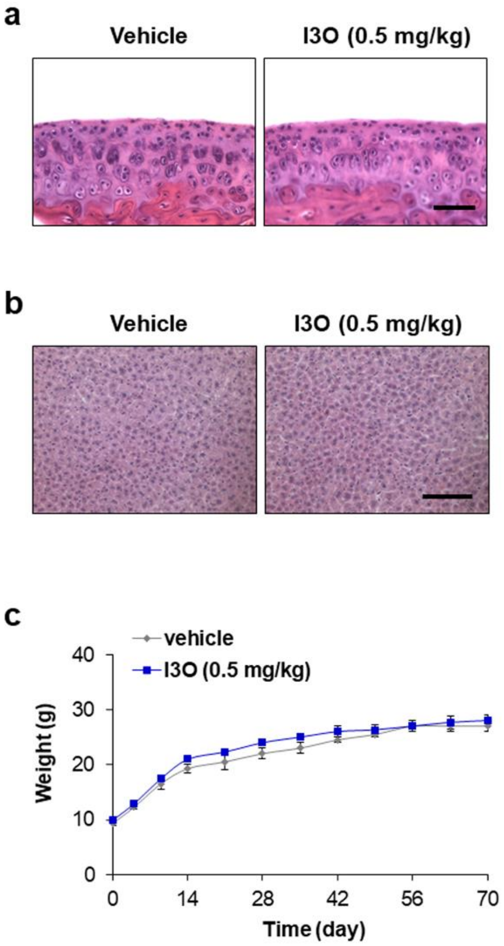

Supplementary Fig. S1. I3O treatment does not cause critical abnormalities in liver, articular cartilage, and weight.

a–c The data shown were obtained from the same mice that were examined in Figure 5. Articular cartilage (**a**) and liver (**b**) tissues were stained with H&E. Throughout I3O treatment, animal weight was measured every 5 or 7 days (mean±s.e.m., n=13 per group) (**c**). Scale bars, 0.1 mm.
